# LINE-1 Methylation Levels in Leukocyte DNA and Risk of Renal Cell Cancer

**DOI:** 10.1371/journal.pone.0027361

**Published:** 2011-11-04

**Authors:** Linda M. Liao, Paul Brennan, Dana M. van Bemmel, David Zaridze, Vsevolod Matveev, Vladimir Janout, Hellena Kollarova, Vladimir Bencko, Marie Navratilova, Neonila Szeszenia-Dabrowska, Dana Mates, Nathaniel Rothman, Paolo Boffetta, Wong-Ho Chow, Lee E. Moore

**Affiliations:** 1 Division of Cancer Epidemiology and Genetics, National Cancer Institute, Bethesda, Maryland, United States of America; 2 International Agency for Research on Cancer, Lyon, France; 3 Institute of Carcinogenesis, Moscow, Russian Federation; 4 Palacky University, Olomouc, Czech Republic; 5 Charles University in Prague, Prague, Czech Republic; 6 Masaryk Memorial Cancer Institute, Bmo, Czech Republic; 7 Institute of Occupational Medicine, Lodz, Poland; 8 Institute of Public Health, Bucharest, Romania; 9 The Tisch Cancer Institute, Mount Sinai School of Medicine, New York, New York, United States of America; 10 International Prevention Research Institute, Lyon, France; The University of Arizona, United States of America

## Abstract

**Purpose:**

Leukocyte global DNA methylation levels are currently being considered as biomarkers of cancer susceptibility and have been associated with risk of several cancers. In this study, we aimed to examine the association between long interspersed nuclear elements (LINE-1) methylation levels, as a biomarker of global DNA methylation in blood cell DNA, and renal cell cancer risk.

**Experimental Design:**

LINE-1 methylation of bisulfite-converted genomic DNA isolated from leukocytes was quantified by pyrosequencing measured in triplicate, and averaged across 4 CpG sites. A total of 328 RCC cases and 654 controls frequency-matched(2∶1) on age(±5years), sex and study center, from a large case-control study conducted in Central and Eastern Europe were evaluated.

**Results:**

LINE-1 methylation levels were significantly higher in RCC cases with a median of 81.97% (interquartile range[IQR]: 80.84–83.47) compared to 81.67% (IQR: 80.35–83.03) among controls (p = 0.003, Wilcoxon). Compared to the lowest LINE-1 methylation quartile(Q1), the adjusted ORs for increasing methylation quartiles were as follows: OR(Q2) = 1.84(1.20−2.81), OR(Q3) = 1.72(1.11−2.65) and OR(Q4) = 2.06(1.34−3.17), with a p-trend = 0.004. The association was stronger among current smokers (p-trend<0.001) than former or never smokers (p-interaction = 0.03). To eliminate the possibility of selection bias among controls, the relationship between LINE-1 methylation and smoking was evaluated and confirmed in a case-only analysis, as well.

**Conclusions:**

Higher levels of LINE-1 methylation appear to be positively associated with RCC risk, particularly among current smokers. Further investigations using both post- and pre-diagnostic genomic DNA is warranted to confirm findings and will be necessary to determine whether the observed differences occur prior to, or as a result of carcinogenesis.

## Introduction

Region-specific hypermethylation and global hypomethylation of DNA are associated with carcinogenesis [1]. Aberrant DNA hypermethylation tends to occur in CpG-rich promoter regions and is associated with transcriptional silencing of genes, such as tumor suppressor genes [2]. In contrast, genome-wide DNA hypomethylation occurs primarily in repetitive sequences of DNA, such as heterochromatic regions and retrotransposons [3]. Several studies have observed an association between methylation levels of leukocyte DNA and risk of bladder, breast, and colorectal cancer [4], [5], [6], [7]. However, the association between global methylation levels and renal cell cancer (RCC) has not yet been evaluated.

Long interspersed nuclear elements (LINE-1) are non-long-terminal-repeat (non-LTR) retrotransposons that make up about 17% of the human genome, with ∼500,000 elements normally dispersed throughout the human genome [3], [8]. LINE-1 elements are typically heavily methylated in normal tissues, while LINE-1 hypomethylation has been reported in cancer tissues [9]. Quantification of CpG methylation within LINE-1 elements is an inexpensive high-throughput assay that has been extensively used as a proxy of global cytosine methylation (5MeC) levels [10], [11]. Several case-control studies have suggested that LINE-1 methylation levels measured in leukocyte DNA could be a potential biomarker of cancer susceptibility and genomic instability; however, the relationship between LINE1 and other biomarkers of global methylation status such as Alu [12] is only beginning to be explored in large well-designed studies of cancer risk [13], [14], [15], [16]. Moreover, relationships between methylation levels in target tissues and non-invasively collected proxy tissue DNA samples are also not well understood.

The incidence of RCC, the most common malignancy of renal cancer, varies worldwide [17], with some of the highest rates occurring in Central and Eastern Europe [18], [19]. Cigarette smoking, obesity, and hypertension are well-established risk factors for RCC, but these account for only an estimated 50% of cases [19]. To explore the potential influence of global methylation as a risk factor for RCC, we compared LINE-1 methylation levels in leukocyte DNA in a subset of RCC cases and controls enrolled in a multi-center case-control study. We examined potential effect modification by common germline polymorphisms and known RCC risk factors. Lastly, *VHL* gene inactivation in RCC tumor tissue is frequently (up to 91%) observed. *VHL* alteration, through either promoter hypermethylation or sequence alterations, is considered an early and frequent event in renal carcinogenesis and has been used as a biomarker of renal tumor heterogeneity. Therefore, LINE-1 methylation levels were evaluated among heterogeneous case subgroups to examine whether risk of having a specific type of *VHL* alteration in tumor DNA might be modified by global methylation levels in genomic DNA.

## Results

A majority of study participants were from the Czech Republic, with a slightly higher proportion among cases. Cases were more likely than controls to have a higher BMI (p = 0.07) and have a lower vegetable intake (p = 0.01; Table 1). Distributions of age, smoking status and self-reported hypertension were comparable between cases and controls.

**Table 1:** LINE-1 Methylation Levels by Characteristics of Study Participants in the Central and Eastern European Renal Cancer Study.

	Cases						Controls						
	N	%	Average	25%	Median	75%	N	%	Average	25%	Median	75%	p-value[Table-fn nt101]
Overall	328		82.13	80.84	81.97	83.47	654		81.74	80.35	81.67	83.03	0.003**
Sex													
Male	203	61.9	82.37	81.09	82.19	83.76	396	60.6	81.97	80.44	81.89	83.32	
Female	125	38.1	81.73	80.51	81.58	82.65	258	39.4	81.40	80.09	81.29	82.72	0.0003
Age													
30–39	10	3.1	82.14	80.57	82.29	82.96	18	2.8	80.98	80.21	80.72	81.34	
40–49	46	14	82.25	81.08	82.09	83.24	99	15.1	81.78	80.27	81.46	83.23	
50–59	101	30.8	82.40	80.95	82.22	83.83	197	30.1	81.70	80.01	81.67	83.16	
60–69	105	32	81.97	80.81	81.72	83.25	209	32	81.68	80.35	81.55	82.98	
70+	66	20.1	81.87	80.52	81.59	83.32	131	20	82.00	80.70	81.98	83.22	0.15
Smoking Status													
Never	148	45.4	81.85	80.46	81.83	83.26	284	43.6	81.80	80.37	81.73	83.09	
Ex smoker ≥2 yrs	69	21.2	82.36	81.08	81.96	83.77	156	23.9	82.00	80.79	81.90	83.00	
Current	109	33.4	82.38	80.95	82.17	83.53	212	32.5	81.48	79.95	81.38	82.96	0.10
BMI													
<25	88	26.8	82.27	80.86	82.26	83.74	213	32.8	81.84	80.25	81.86	83.05	
25–30	148	45.1	82.04	80.76	81.96	83.23	292	44.9	81.72	80.35	81.56	83.06	
≥30	92	28.1	82.13	80.91	81.86	83.43	145	22.3	81.66	80.39	81.63	82.98	0.38
Vegetables (Tertile)													
1-Low	90	28.2	82.41	81.02	82.21	83.84	156	24.6	81.58	80.17	81.55	82.98	
2-Medium	137	42.9	82.19	80.87	82.05	83.51	234	36.9	81.90	80.39	81.80	83.23	
3-High	92	28.8	81.75	80.44	81.66	82.93	244	38.5	81.62	80.11	81.49	82.78	0.99
Self-Reported Hypertension													
No	184	56.1	82.14	80.80	81.95	83.53	404	61.8	81.62	80.27	81.46	82.86	
Yes	144	43.9	82.11	80.86	82.04	83.42	250	38.2	81.95	80.41	82.03	83.23	0.04
Family History of Cancer													
No Cancer	214	65.2	82.15	80.80	82.01	83.65	473	72.3	81.78	80.38	81.66	83.04	
Has Kidney Cancer	11	3.4	82.45	81.82	82.29	82.94	8	1.2	82.18	80.97	82.68	84.09	
Has other Cancer	103	31.4	82.03	80.81	81.91	83.37	173	26.5	81.63	80.15	81.67	82.97	0.43
Center													
Romania	42	12.8	81.37	80.26	81.21	82.23	86	13.2	81.47	80.13	81.36	87.11	
Poland	43	13.1	82.59	81.30	82.29	83.87	116	17.7	81.91	80.05	81.66	87.74	
Russia	38	11.6	83.05	81.72	82.84	84.54	120	18.4	81.06	79.78	80.90	85.43	
Czech Republic	205	62.5	82.01	80.75	81.91	83.24	332	50.8	82.00	80.74	81.98	88.35	0.01

*p-value for potential determinants analysis derived from unadjusted linear regression models among controls only.

**p-value for overall comparison between cases and controls derived from Wilcoxon signed rank test.

Using linear regression models, we evaluated the contribution of selected participant characteristics hypothesized to be associated with LINE-1 methylation levels among controls (n = 654; Table 1). Among controls, males had statistically significantly higher LINE-1 methylation levels (median = 81.89%) than females (median = 81.29%; p = 0.0003). Individuals who reported having hypertension were also associated with statistically significant higher LINE-1 methylation levels (median = 82.03%) than those that did not (median = 81.46%; p = 0.04). A significant difference among centers was also detected among controls (p = 0.01), which we will adjust for in our models. No distinct trends in LINE-1 methylation levels by age, smoking status, BMI or vegetable intake were detected among controls. The distribution of LINE-1 %5MeC among cases is also provided in Table 1 for comparison.

Overall, median LINE-1 methylation levels were significantly higher in RCC cases 81.97% (interquartile range: 80.84–83.47) compared to 81.67% (interquartile range: 80.35–83.03) among controls (p = 0.003, Wilcoxon; Table 1). Compared with individuals in the lowest LINE-1 methylation quartile, individuals in the highest quartile were associated with a 2-fold increased risk of RCC (p for trend = 0.004; Table 2). The adjusted OR for RCC in association with a 1% increase in overall LINE-1 methylation was 1.10 (95% CI: 1.03–1.19; p = 0.008). Methylation varied slightly across each of the four CpG sites sequenced, with the lowest median %5MeC observed at the fourth locus (median of cases: 78.4%, controls: 78.2%) and the highest levels on the first locus (median of cases: 85.4%, controls: 84.8%). Compared to controls, cases had statistically significantly higher %5MeC at each locus (p-values ranging from <0.0001 to 0.05, Wilcoxon). Higher %5MeC at locus 1 demonstrated the strongest association with risk of RCC (OR = 1.24, 95% CI: 1.14–1.35); however, similar upward trends were observed at the other three loci (locus 2: OR = 1.07, 95% CI: 0.99–1.14; locus 3: OR = 1.06, 95% CI: 1.00–1.13; locus 4: OR = 1.05, 95% CI: 0.99–1.11).

**Table 2:** Association between LINE-1 Methylation Levels and Risk of Renal Cell Cancer.

Quartile	LINE-1 position 1				LINE-1 position 2				LINE-1 position 3				LINE-1 position 4				LINE-1 overall			
	Range	Cases/Controls	OR	95% CI	Range	Cases/Controls	OR	95% CI	Range	Cases/Controls	OR	95% CI	Range	Cases/Controls	OR	95% CI	Range	Cases/Controls	OR	95% CI
Q1	78.4–83.6	39/163	1.00		73.8–80.7	55/163	1.00		69.6–79.8	57/164	1.00		70.5–76.6	58/164	1.00		74.5–80.3	47/162	1.00	
Q2	83.6–84.8	66/164	**1.74**	**1.09–2.77**	80.7–82.1	97/164	**1.58**	**1.05–2.39**	79.8–81.5	89/163	1.48	0.98–2.24	76.6–78.2	87/163	1.44	0.96–2.18	80.3–81.7	93/165	**1.84**	**1.20–2.81**
Q3	84.8–86.0	107/164	**2.75**	**1.77–4.28**	82.1–83.6	88/164	1.43	0.95–2.17	81.5–83.2	90/164	1.46	0.97–2.20	78.2–80.0	87/164	1.42	0.95–2.14	81.7–83.0	86/164	**1.72**	**1.11–2.65**
Q4	86.0–90.2	116/163	**3.09**	**1.99–4.81**	83.7–89.7	88/163	1.44	0.95–2.19	83.2–90.1	92/163	1.58	1.04–2.39	80.1–88.3	96/163	**1.56**	**1.04–2.36**	83.0–88.8	102/163	**2.06**	**1.34–3.17**
P-trend				**<0.0001**				0.19				0.05				**0.05**				**0.004**
																				
**Continuous**			**1.24**	**1.14–1.35**			1.07	0.99–1.14			**1.06**	**1.00–1.13**			1.05	0.99–1.11			**1.10**	**1.03–1.19**

Adjusted for sex, age, center, tobacco status, BMI, high blood pressure and vegetable intake.

**Figure 1:**
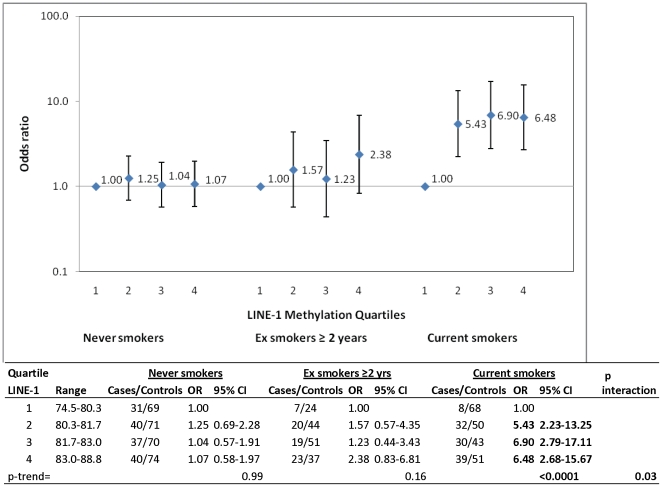
Association between LINE-1 methylation levels and risk of RCC stratified by smoking status. Odds ratios and 95% confidence intervals for the association between LINE-1 methylation levels and RCC, adjusted for sex, age, center, BMI, high blood pressure and vegetable intake.

In analyses stratified by smoking status, the increased risk with the highest LINE-1 %5MeC quartile (Q4) was more pronounced among current smokers (OR_Q4_ = 6.48, 95% CI: 2.68–15.67; p-trend <0.0001) than among former or never smokers (p-interaction = 0.03; Figure 1). A positive association with RCC risk was similarly restricted to current smokers across individual loci (Table S1). To further explore the interaction between smoking and high LINE-1 %5MeC (Q2–Q4), we conducted a case-only analysis to ensure that the association was not due to a control series that was not representative of the study population with regard to smoking status. Compared to RCC cases that were never smokers, positive departures from multiplicativity were observed for RCC cases that were either former (IOR = 1.83, 95% CI: 0.69–4.83) or current smokers (IOR = 2.47, 95% CI: 0.91–6.70; p-trend = 0.06). This would indicate that the relationship of increased global methylation and RCC is stronger among current smokers than never smokers. In both analyses, smoking status and LINE-1 methylation levels appeared to modify the contribution of the other risk factor. Additional stratified analyses by sex, BMI, vegetable intake, and self-reported hypertension did not result in any significant differences (data not shown).

To evaluate whether common variation in selected genes involved in one-carbon and tobacco metabolism modified the observed association, we conducted stratified analyses by seven functional variants in five genes (*MTHFR, MTR, TYMS, GSTM1,* and *GSTTI*). Two genetic variants, *MTHFR* c.1298A>C and *MTR c.2756A>G,* appeared to influence the association between LINE-1 methylation levels and RCC risk (Table 3). We observed a strong positive trend between LINE-1 methylation quartiles and RCC risk among those that carried at least one copy of the C minor allele of *MTHFR* c.1298A>C (p-trend = 0.0009; p-interaction = 0.15). Increased risk with higher levels of LINE-1 methylation was restricted to those with the AA genotype of *MTR c.2756A>G* (p-trend = 0.004; p-interaction = 0.19) and was not significant among those carrying at least one copy of the minor allele. Neither of these variants demonstrated statistically significant main effects, which has been previously published [20].

**Table 3:** Modification of Association between LINE-1 methylation and RCC risk by selected polymorphisms.

Quartile	Range	Cases/Controls	OR	95% CI	Cases/Controls	OR	95% CI	p interaction
MTHFR- A1298C, Ex8-62A>C, E429A, rs1801131								
		AA			AC/CC			
1	74.5–80.3	25/63	1.00		20/93	1.00		
2	80.3–81.7	39/65	1.46	0.77–2.78	50/92	**2.50**	**1.35–4.66**	
3	81.7–83.0	32/70	1.06	0.55–2.06	48/81	**2.81**	**1.50–5.28**	
4	83.0–88.8	37/65	1.55	0.80–3.01	46/66	**3.16**	**1.65–6.03**	
P trend =				0.38			**0.0009**	0.15
MTR- A2756G, Ex26-20A>G, D919G, rs1805087								
		AA			AG/GG			
1	74.5–80.3	26/101	1.00		20/56	1.00		
2	80.3–81.7	57/99	**2.22**	**1.27–3.89**	32/62	1.21	0.60–2.43	
3	81.7–83.0	58/90	**2.33**	**1.32–4.10**	23/67	0.89	0.42–1.87	
4	83.0–88.8	56/90	**2.47**	**1.40–4.37**	35/48	1.99	0.95–4.17	
P trend =				**0.004**			0.14	0.19

Adjusted for sex, age, center, tobacco status, BMI, high blood pressure and vegetable intake.

Because *VHL* gene inactivation is a frequent early event in RCC that has been used to define molecular subgroups of renal cancers [21], we explored whether global methylation was associated with a particular subset of RCC cases defined by the mechanism through which *VHL* gene inactivation in tumor DNA occurs. We defined RCC subgroups as having *VHL* gene inactivation through epigenetic mechanisms (promoter hypermethylation), genetic mechanisms (changes in coding sequence that would result in an altered protein (i.e. missense mutations, deletions, insertions, splice site mutations) or wild type tumors (those without observable *VHL* inactivation). Among a subset of RCC cases on which data was available on *VHL* promoter methylation or genetic alteration status in somatic tissue (n = 144), the risk of having an alteration in *VHL* increased with increasing quartiles of methylation (OR_Q2_ = 1.26, 95%CI:0.34–4.64; OR_Q3_ = 1.62, 95%CI:0.42–6.18; and OR_Q4_ = 2.57, 95%CI:0.68–9.72; p-trend = 0.12). Although suggestive, more cases are needed to fully evaluate this association.

## Discussion

This study assessed the role of LINE-1 methylation in peripheral blood DNA as a proxy for global methylation in renal tumor tissue. In our study, higher LINE-1 %5MeC was associated with an increased risk of RCC. Higher LINE-1 %5MeC levels appeared to be a stronger risk factor for the development of RCC in current smokers than in former or never smokers. In addition, results from our study suggest a possible interaction between LINE-1 methylation and *MTHFR* c.1298A>Cand *MTR c.2756A>G* with RCC.

The association we observed contrasts with what has been observed in other cancer epidemiologic studies. Global DNA hypomethylation in tumor tissue has long been observed, but varies widely within and between types of cancer [7], [22]. Global DNA hypomethylation of leukocyte DNA, measured by %5MeC content, has been associated with increased cancer risk in several recent case-control studies, including a large case-control study of bladder cancer [4], [6], [7]. In several case-control studies, lower levels of LINE-1 has been associated with increased risk of testicular tumors [14], and gastric [13], head and neck [16], and bladder cancer [15]. However, a small study of breast cancer found no difference in %5MeC LINE-1 levels between cases and controls, but a significantly lower %5MeC levels among cases [5]. This same study also noted that levels of %5MeC and %5MeC LINE-1 (both measured in leukocyte DNA) did not correlate well [5].

Limited data are available on the impact of global DNA methylation levels in renal tumors and are difficult to compare due to different methods of assessing methylation. To the best of our knowledge, data are not available on the correlation between global methylation status in serum and renal tissue. Among promoter methylation studies, one study reported that the promoter methylation state of *Wnt* genes measured in serum generally matched that of corresponding renal tumor samples [23], while the proportion of cases where promoter methylation of specific cancer-related genes detected in serum matched with methylation of the same marker in tumors ranged from 0 to 60% [24]. A few studies have detected increased global methylation in RCC tissue. Arai et al. reported that genome-wide DNA methylation profiles, including global hypermethylation, determined from adjacent normal renal tissue were highly associated with the aggressiveness of the corresponding RCC [25]. A small study by Minardi et al. noted a significant increase in global methylation in tumor tissues with increasing grade of RCC [26]. Two experimental studies have described *LINE*-1 methylation status in RCC tissue samples. Chalitchagorn et al. demonstrated that LINE-1 methylation varied significantly among different tissue types, but observed no difference in *LINE*-1 hypomethylation levels between RCC and renal tissues [9]. Another study observed an absence of hypomethylation of LINE-1 sequences across different stages and grades of RCC, which was in contrast to other urothelial carcinomas [27]. Together, these studies suggest that RCC may lack the typical association with DNA methylation observed in other cancers; however, given the varying assessment methods used, further studies are needed.

To the best of our knowledge, this is the first study to find an association with higher rather than lower LINE-1 methylation levels (measured in leukocyte DNA) and cancer risk. Phokaew et al. observed sporadic instances of hypermethylation in LINE-1 loci among cancer cell lines[28]. Specific hypermethylation of LINE-1 has also been reported in the abnormal overgrowth and differentiation of placenta tissue [29]. Although LINE-1 methylation levels are expected to provide a quick and easy assessment of global methylation, only a third of genomic DNA methylation is estimated to occur in repetitive elements [11]. It is also possible that RCC has an unusual relationship with methylation within repetitive elements compared to other types of cancer. Lastly, until our findings are replicated there also remains the possibility that the association we observed could be due to chance. The observed association appears somewhat robust and consistent across our analyses suggesting that these findings were not due to chance; however, this finding does require replication in a second study population.

In our evaluation of predictors of LINE-1 methylation levels in leukocyte DNA, sex was the only characteristic that was strongly associated with variation in levels. Males had significantly higher LINE-1 methylation levels than females, which is in agreement with other studies [14], [15], [16], [30], [31], [32], [33]. Self-reported hypertension was also associated with higher LINE-1 methylation levels, but has not been previously evaluated in published studies. Age is generally considered to be inversely associated with increased global methylation [34]; however, age was not correlated with LINE-1 methylation levels in our study or in several previous studies [9], [16], [31], [32], [35], [36]. Smoking has been associated with both promoter hypermethylation and genomic hypomethylation in tumor DNA [16], [37]. However, we did not observe an association between smoking status and LINE-1 methylation levels in leukocyte DNA, which is consistent with results from other studies [15], [16], [31], [32]. The lack of association between LINE-1 methylation levels in blood with various characteristics in our study was generally similar to what has been observed in other studies; however, it should be noted that our controls may not be representative as they were hospital-based controls.

Smoking has been previously linked to global methylation levels in tumor tissue [16], [37]. Based on data from our study and others, however, there does not seem to be a well-defined relationship with regards to genomic DNA isolated from blood [15], [16], [31], [32]. Given the conflicting data on the association between smoking and methylation levels, it is possible that the effect of smoking carcinogens on methylation status may be different among different tissue types. In our data, the risk associated with higher %5MeC of LINE-1 appears to be enhanced in current smokers, suggesting an interaction between smoking and LINE-1 hypermethylation. It should be noted that previous reports from this study population did not observe a smoking main effect, presumably due to some source of control selection bias [38]. However, we did notice that among the three highest quartiles of LINE-1, current smoking was associated with an increase in RCC risk compared to never smokers. To deal with the bias, we conducted a case-only analysis which minimizes any potential selection bias from the use of hospital-based controls. These results among cases were similar and confirmed that current smokers who are heavily methylated within LINE-1 have a higher risk of RCC than never smokers who are heavily methylated. Several potential mechanisms for how smoking affects DNA methylation status exist, such as indirectly through increased systemic inflammation or double strand-break damage, or directly through the altered expression of DNA methyltransferases, but the exact mechanism is poorly understood [39].

As genes involved in one-carbon metabolism have been associated with global methylation levels and RCC, we conducted exploratory analyses using available genotype data to assess whether polymorphisms modified the association between LINE-1 methylation levels and RCC risk. In the present study, results from two genetic variants *MTHFR* c.1298A>Cand *MTR c.2756A>G* were suggestive. Other studies of global methylation and cancer have not observed any noticeable differences in cancer risk by these variants [4], [13], [15].

To our knowledge, this is the first study to evaluate LINE-1 methylation levels in leukocyte DNA and risk of RCC. Although a subset of the full case-control study was selected, this study has sufficient statistical power to detect associations and they were not different to the group as a whole. A limitation is that this case-control study included hospital-based controls and DNA from cases was collected before treatment yet post-diagnosis. We did not detect substantial differences in LINE-1 methylation levels by disease groups among controls, or by stage and grade of cases. This suggests that disease progression is less likely to have affected our results. The case-only analysis conducted, eliminated the potential selection bias that occurs with the use of hospital-based controls, and supported the association observed in the stratified analysis. Although LINE-1 methylation levels are considered to be a proxy for global DNA methylation overall, the two measures are not entirely interchangeable and could be measuring different aspects of global methylation. Regardless, many studies have demonstrated that use of LINE-1 methylation levels in blood can provide a useful marker for disease and would offer a simple, noninvasive method to identify individuals at risk for RCC.

In summary, our findings suggest that LINE-1 methylation levels in leukocyte DNA are positively associated with risk of RCC and may serve as a biomarker for RCC susceptibility. Investigations to determine whether global methylation in genomic DNA reflects epigenetic alterations in renal tissue are necessary to further elicit the role of epigenetics and RCC. To address these questions, future studies in prospectively collected DNA samples will be necessary, as well as studies designed to address the apparent interaction between methylation status and tobacco smoking related to RCC risk.

## Materials and Methods

### Ethics Statement

The study protocol was approved by relevant ethics committees and institutional review boards of all participating centers, the International Agency for Research on Cancer (IARC), and the U.S. National Cancer Institute (NCI) at the U.S. National Institutes of Health. All study subjects and their physicians provided written informed consent.

### Study Population

The Central and Eastern European Renal Cancer Study (CEERCC) is a hospital-based case-control study of renal cancer (1,097 cases and 1,555 controls) that was conducted in seven centers in Eastern and Central Europe (Moscow, Russia; Bucharest, Romania; Lodz, Poland; and Prague, Olomouc, Ceske Budejovice and Brno, Czech Republic). The study population has been previously described [40]. Briefly, newly diagnosed and histologically confirmed cases of renal cancer but not renal pelvis (ICD-0-2 code C64) between the ages of 20 and 79 years were recruited from 1999 through 2003. Trained medical staff reviewed medical records and extracted information on date and method of diagnosis, histological classification, tumor stage and grade. Eligible controls were chosen from patients admitted to the same hospital as cases for conditions unrelated to smoking or genitourinary disorders (except for benign prostatic hyperplasia) and were frequency-matched to cases on age, sex, and study center. No single disease made up more than 20% of the control group. All recruited cases and controls were Caucasian. Response rates at each center ranged from 90.0 to 98.6% for cases and from 90.3 to 96.1% for controls. Standardized lifestyle and food frequency questionnaires were administered in person by trained personnel [38], [41].

Blood samples were collected and stored at −80°C and subsequently shipped to the National Cancer Institute (NCI). Genomic DNA was extracted from buffy coat by the standard phenol chloroform method at the NCI laboratory. To evaluate global methylation, we selected a subset of RCC cases and controls with a large amount of available DNA (≥10 µg) and data on *VHL* status using the following matching criteria. Controls were randomly selected and frequency-matched (2∶1) on age (±5 years), sex and study center (if possible) to obtain sufficient power (90% to detect a minOR of 1.80). All subjects in this study provided written informed consent. This study was approved by the institutional review boards at the NCI, International Agency for Research on Cancer (IARC), and each participating center.

### Genotyping

Genotyping assays were performed at NCI's Core Genotyping Facility. The following functional single nucleotide polymorphisms (SNPs) within three genes were considered in this study because of their role in providing precursors needed for DNA synthesis and repair, as well as DNA methylation: 5,10 methylenetetrahydrofolate reductase (*MTHFR*; c.1298A>C, *c.677C>T)*, 5-methyltetrahydrofolate-homocysteine methyltransferase (*MTR; c.2756A>G*), and thymidylate synthetase (*TYMS; Ex8+157C>T, Ex8+227A>G*). Glutathione S-transferase mu 1 (*GSTM1*) and glutathione S-transferase theta 1 (*GSTT1*) deletions were also selected because of previous associations with risk of RCC and their role in phase II metabolism. Specific experimental methods have been described previously (http://snp500cancer.nci.nih.gov) [20], [42].

### Quantification of LINE-1 Methylation Levels

LINE-1 methylation levels were quantified using a pyrosequencing assay at EpigenDx (Worcester, MA) [43], [44], [45]. Our assay is designed to examine the methylation status at four CpG sites in the promoter of the LINE-1 region (GenBank Accession #: M80343, −605, −593, −590 and −583 bp from ATG of ORF1) [16], [46]. Briefly, 500 ng of leukocyte DNA was bisulfate treated and purified using the Zymo DNA Methylation Kit (Zymo research, Orange, CA). Bisulfate treated DNA was purified and eluted in 20 µL elution buffer. Each 50ul PCR contained 10X PCR buffer, 3.0 mM MgCl_2_, 200 µM dNTPs, 0.2 µM primers, 1.25 U DNA polymerase (HotStar, Qiagen Inc., Alameda, CA) 1.25 U, and ∼10 ng of bisulfite converted DNA. The polymerase was activated by incubation at 95°C for 10 min followed by 34 cycles of 95°C for 15sec, 45°C for 30 sec and 72°C for 30 sec. The reaction was then allowed to extend for 5 min at 72°C. A universal biotinylated primer was used in the initial PCR reaction to allow for isolation of the amplicon, followed by denaturation and release of a single strand product for pyrosequencing [47]. PCR products (10 µl) were sequenced using the Pyrosequencing PSQ96 HS System (Pyrosequencing Qiagen) following the manufacturer's instructions (Pyrosequencing Qiagen). Methylation status at each of 4 loci was analyzed individually as a T/C SNP using QCpG software (Pyrosequencing Qiagen). Methylation status at all four loci are averaged together to provide an overall percent 5MeC status. A sample pyrogram is included as supplementary data (Figure S1). Percent DNA methylation within LINE-1 was measured in triplicate.

### Statistical Analyses

Triplicate LINE-1 5MeC measurements were averaged together. Individual runs with >7.5% bisulfite unconverted cytosine values and samples with a coefficient of variation (CV) >10% were excluded from our analyses (n = 19). We conducted a sensitivity analysis, excluding individuals with CV >5%. This exclusion did not result in a significant difference in risk estimates by at least 10% and thus these subjects remained in the analyses. After these exclusions, data were available on 982 samples (328 RCC cases and 654 controls). Differences between cases and controls were tested for significance using Wilcoxon signed rank tests. To identify potential determinants of LINE-1 methylation levels and/or factors that could modify the association between LINE-1 methylation levels and renal cancer risk, linear regression models were used to evaluate differences among controls in relation to selected characteristics. The distribution of LINE-1 mean %5MeC among controls was used to determine cut points for quartiles. To assess the association between LINE-1 methylation levels and RCC, logistic regression models, adjusted for the matching factors, tobacco status, BMI, vegetable intake and self-reported hypertension, were used to estimate odds ratios(OR) and 95% confidence intervals(95% CI). Tests for trend were calculated by modeling a variable coded 0, 1, 2 and 3 for each quartile. We also modeled LINE-1 as a continuous value. To evaluate potential effect modification of LINE-1 methylation, stratified analyses by smoking status, sex, BMI, vegetable intake, self-reported hypertension and selected SNPs were conducted. Common functional variants within *MTHFR, MTR, TYMS, GSTM1,* and *GSTT1* were evaluated for association with LINE-1 methylation. The homozygous major allele was coded as the referent group, and the heterozygous and homozygous minor allele genotypes were combined together due to small numbers of subjects homozygous for the minor alleles. A case-only analysis evaluating the interaction between smoking and LINE-1 methylation levels was also conducted to provide an estimate of effect modification (interaction OR: IOR) that would not be influenced by selection biases among our controls. The IORs represent the departure of the joint effect of smoking and LINE-1 methylation from that expected under a multiplicative model on RCC risk. All analyses were conducted using SAS version 9.1. (SAS Institute, Cary, NC).

## Supporting Information

Figure S1 **Sample pyrogram demonstrating LINE-1 methylation levels.**
(DOC)

Table S1 **Stratified Analyses by Smoking Status and Individual LINE-1 position.** Odds ratios and 95% confidence intervals for the association between LINE-1 methylation levels and RCC, adjusted for sex, age, center, BMI, high blood pressure and vegetable intake.
(DOC)
