# Supplementary material for: LINE-1 Methylation Levels in Leukocyte DNA and Risk of Renal Cell Cancer
Source: PLoS One. 2011 Nov 4;6(11):e27361. doi: 10.1371/journal.pone.0027361 (PMC3208631; doi:10.1371/journal.pone.0027361)
Supplement: Figure S1 — Sample pyrogram demonstrating LINE-1 methylation levels. (DOC) [file pone.0027361.s001.doc]

**Supplementary Figure 1. Sample pyrogram demonstrating LINE-1 methylation levels**
