# Supplementary material for: LINE-1 Methylation Levels in Leukocyte DNA and Risk of Renal Cell Cancer
Source: PLoS One. 2011 Nov 4;6(11):e27361. doi: 10.1371/journal.pone.0027361 (PMC3208631; doi:10.1371/journal.pone.0027361)
Supplement: Table S1 — Stratified Analyses by Smoking Status and Individual LINE-1 position. Odds ratios and 95% confidence intervals for the association between LINE-1 methylation levels and RCC, adjusted for sex, age, center, BMI, high blood pressure and vegetable intake. (DOC) [file pone.0027361.s002.doc]

**Table S1. Stratified Analyses by Smoking Status** and Individual LINE-1 position

|  | **Quartile** | **Never smoker** | | | **Ex smoker ≥2 yrs** | | | **Current smoker** | | |  |
| --- | --- | --- | --- | --- | --- | --- | --- | --- | --- | --- | --- |
| **LINE-1 position 1** |  | **Cases/Controls** | **OR** | **95% CI** | **Cases/Controls** | **OR** | **95% CI** | **Cases/Controls** | **OR** | **95% CI** | **p interaction** |
|  | 1 | 22/74 | 1.00 |  | 9/28 | 1.00 |  | 7/61 | 1.00 |  |  |
|  | 2 | 38/63 | **2.18** | **1.14-4.15** | 8/47 | 0.43 | 0.14-1.33 | 20/53 | **3.22** | **1.23-8.46** |  |
|  | 3 | 43/71 | **1.91** | **1.02-3.60** | 22/39 | 1.79 | 0.69-4.64 | 41/54 | **6.72** | **2.70-16.75** | |
|  | 4 | 45/76 | **1.97** | **1.05-3.70** | 30/42 | **2.73** | **1.06-7.04** | 41/44 | **8.12** | **3.19-20.64** | |
|  | P trend= |  |  | 0.08 |  |  | **0.001** |  |  | **<0.0001** | **0.01** |
| **LINE-1 position 2** | |  |  |  |  |  |  |  |  |  |  |
|  | 1 | 25/62 | 1.00 |  | 15/26 | 1.00 |  | 15/74 | 1.00 |  |  |
|  | 2 | 50/78 | 1.33 | 0.72-2.46 | 15/43 | 0.69 | 0.28-1.70 | 30/43 | **3.95** | **1.81-8.62** |  |
|  | 3 | 36/73 | 1.02 | 0.54-1.92 | 20/51 | 0.68 | 0.29-1.62 | 32/40 | **4.53** | **2.07-9.89** |  |
|  | 4 | 37/71 | 1.04 | 0.55-2.00 | 19/36 | 0.92 | 0.38-2.22 | 32/55 | **2.98** | **1.41-6.34** |  |
|  | P trend= |  |  | 0.77 |  |  | 0.88 |  |  | **0.008** | 0.06 |
| **LINE-1 position 3** | |  |  |  |  |  |  |  |  |  |  |
|  | 1 | 32/68 | 1.00 |  | 10/28 | 1.00 |  | 14/67 | 1.00 |  |  |
|  | 2 | 42/72 | 1.26 | 0.70-2.28 | 20/43 | 1.43 | 0.57-3.60 | 26/48 | 2.19 | 0.99-4.85 |  |
|  | 3 | 37/73 | 0.93 | 0.51-1.71 | 18/46 | 1.06 | 0.41-2.71 | 35/45 | **3.93** | **1.84-8.37** |  |
|  | 4 | 37/71 | 1 | 0.54-1.85 | 21/39 | 1.75 | 0.66-4.67 | 34/52 | **2.98** | **1.40-6.37** |  |
|  | P trend= |  |  | 0.73 |  |  | 0.43 |  |  | **0.002** | 0.10 |
| **LINE-1 position 4** | |  |  |  |  |  |  |  |  |  |  |
|  | 1 | 36/71 | 1.00 |  | 9/27 | 1.00 |  | 13/65 | 1.00 |  |  |
|  | 2 | 37/66 | 1.08 | 0.60-1.96 | 21/43 | 1.56 | 0.61-3.99 | 28/54 | **2.59** | **1.17-5.75** |  |
|  | 3 | 38/75 | 0.95 | 0.53-1.69 | 16/47 | 1.01 | 0.38-2.68 | 33/41 | **4.30** | **1.95-9.52** |  |
|  | 4 | 37/72 | 0.88 | 0.48-1.60 | 23/39 | 2.00 | 0.76-5.26 | 35/52 | **3.43** | **1.58-7.46** |  |
|  | P trend= |  |  | 0.59 |  |  | 0.31 |  |  | **0.001** | 0.06 |
